# Identification of a Raloxifene Analog That Promotes AhR-Mediated Apoptosis in Cancer Cells

**DOI:** 10.3390/biology6040041

**Published:** 2017-12-01

**Authors:** Hyo Sang Jang, Martin Pearce, Edmond F. O’Donnell, Bach Duc Nguyen, Lisa Truong, Monica J. Mueller, William H. Bisson, Nancy I. Kerkvliet, Robert L. Tanguay, Siva Kumar Kolluri

**Affiliations:** 1 Cancer Research Laboratory, Department of Environmental and Molecular Toxicology, Oregon State University, Corvallis, OR 97331, USA; janghy@oregonstate.edu (H.S.J.); pearcem@oregonstate.edu (M.P.); eodonnel@mail.einstein.yu.edu (E.F.O.); nguyebac@oregonstate.edu (B.D.N.); muellemo@oregonstate.edu (M.J.M); 2 Department of Environmental and Molecular Toxicology, Environmental Health Sciences Center, Oregon State University, Corvallis, OR 97331, USA; lisa.truong@oregonstate.edu (L.T.); nancy.kerkvliet@oregonstate.edu (N.I.K.) robert.tanguay@oregonstate.edu (R.L.T.); 3 Department of Environmental and Molecular Toxicology, Oregon State University, Corvallis, OR 97331, USA; bissonw@science.oregonstate.edu; 4 Linus Pauling Institute, Oregon State University, Corvallis, OR 97331, USA; 5 Center for Genome Research and Biocomputing, Oregon State University, Corvallis, OR 97331, USA

**Keywords:** aryl hydrocarbon receptor, raloxifene, Y134, estrogen receptor modulator, hepatoma, agonist, antagonist, anti-cancer drug, triple negative breast cancer, apoptosis

## Abstract

We previously reported that raloxifene, an estrogen receptor modulator, is also a ligand for the aryl hydrocarbon receptor (AhR). Raloxifene induces apoptosis in estrogen receptor-negative human cancer cells through the AhR. We performed structure–activity studies with seven raloxifene analogs to better understand the structural requirements of raloxifene for induction of AhR-mediated transcriptional activity and apoptosis. We identified Y134 as a raloxifene analog that activates AhR-mediated transcriptional activity and induces apoptosis in MDA-MB-231 human triple negative breast cancer cells. Suppression of AhR expression strongly reduced apoptosis induced by Y134, indicating the requirement of AhR for Y134-induced apoptosis. Y134 also induced apoptosis in hepatoma cells without having an effect on cell cycle regulation. Toxicity testing on zebrafish embryos revealed that Y134 has a significantly better safety profile than raloxifene. Our studies also identified an analog of raloxifene that acts as a partial antagonist of the AhR, and is capable of inhibiting AhR agonist-induced transcriptional activity. We conclude that Y134 is a promising raloxifene analog for further optimization as an anti-cancer agent targeting the AhR.

## 1. Introduction

The aryl hydrocarbon receptor (AhR) is a ligand-activated transcription factor that has been well studied in the fields of toxicology, physiology, and pathology [1]. The AhR belongs to the basic helix-loop-helix (bHLH)–Per-Arnt-Sim (PAS) family of proteins, and the bHLH–PAS domain is required for DNA binding and heterodimer formation with other PAS domain proteins such as Arnt [2,3,4]. Members of this family control physiological processes including cancer cell growth and survival, and certain proteins with the bHLH–PAS domain have defined ligand binding pockets that present an opportunity to develop compounds to modulate these desirable biological activities [5].

Our understanding of the AhR and its role in carcinogenesis continues to evolve, and has recently started to receive a great deal of interest as a molecular target for anti-cancer drugs [6,7,8]. The AhR has tumor suppressive functions, and can be activated by distinct ligands with diverse underlying mechanisms of action including inhibition of cell cycle progression and cancer cell survival [9,10]. Specifically, the AhR inhibits cell cycle progression in some cell types by inducing cell cycle inhibitor and tumor suppressor proteins such as p27^Kip1^ [10]. Using small molecule screening approaches, we have previously identified compounds with anti-cancer effects that function through the AhR [11,12,13]. For example, we showed the selective estrogen receptor modulator (SERM) raloxifene is an AhR ligand that induces apoptosis in breast cancer cells in an AhR dependent manner [14,15]. Together, these studies have identified a new class of molecules capable of modulating AhR activity.

The aims of the present study were two-fold. First, we used analogs of raloxifene to investigate the structural aspects of raloxifene needed for AhR activation. Specifically, in order to better understand how raloxifene activates the AhR, we tested different raloxifene analogs for their ability to induce AhR-mediated transcription. We identified two hydroxyl groups on 2-(4-hydroxyphenyl)benzo[b]thiophene-6-ol and the benzothiophene core of raloxifene as important motifs needed for AhR transcriptional activity. On the other hand, the piperidine-containing non-benzothiophene moiety of raloxifene was unable to induce AhR transcriptional activity on its own, whereas substitution of the piperidine ring with 1-isopropylpiperazine rescued some aspects of AhR transcriptional activity.

The second aim of this study was to identify new AhR ligands based on raloxifene and characterize their phenotypic effects in cancer cells. We present data that the 4-hydroxyphenyl benzothiophene core is important for activating the AhR, and is critical for AhR-mediated raloxifene-induced anti-cancer effects. In addition, we found that raloxifene and its analog Y134 inhibit hepatoma cells in an AhR dependent manner. As AhR ligands have long been associated with developmental abnormalities, we also studied the deleterious effects of raloxifene in a zebrafish embryo model. While some toxicity was noted at higher concentrations of raloxifene in the zebrafish embryo model, the toxicity associated with analog Y134 was significantly reduced. In addition, both raloxifene and Y134 inhibited human triple negative breast cancer (TNBC) cells by inducing apoptosis in an AhR dependent manner. Together, these data suggest that optimization of the raloxifene structure is feasible for identifying and developing AhR ligands with improved anti-cancer efficacy.

## 2. Materials and Methods

### 2.1. Cell Lines

All cells were cultured in DMEM (Corning, Manassas, VA, USA) supplemented with 10% FBS (VWR Life Science, Radnor, PA, USA), 100 U/mL penicillin, and 100 mg/mL streptomycin (Corning). All cells were grown at 37 °C in a humidified 5% CO_2_ atmosphere and were subcultured twice per week using trypsin/EDTA.

### 2.2. Generation of AhR Knock Out Cells

AhR knockout was performed as previously reported [16] with modifications. Briefly, MDA-MB-231 cells were seeded at a density of 1 × 10^6^ cells per well in 6-well plates and incubated overnight. Cells were transfected the next day at 80–90% confluencey with 2.5 µg of the indicated plasmid DNA using Lipofectamine 2000 transfection reagent (Thermo Fisher Scientific, Waltham, MA, USA). The transfected cells were selected by culturing in medium containing 2 µg/mL puromycin. Puromycin was removed at least five days before the cells were used for any experiments. The AhR targeting CRISPR-Cas9 vectors used in this study were obtained from GenScript (Piscataway, NJ, USA). pLentiCRISPR v2 control vector was a gift from Feng Zhang (Addgene plasmid # 52961). The three guide RNA sequences used in this study were as follows: AhR-1, 5′-AAGTCGGTCTCTATGCCGCT-3′; AhR-2, 5′-TTGCTGCTCTACAGTTATCC-3′; AhR-3, 5′-AATTTCAGCGTCAGCTACAC-3′.

### 2.3. Chemicals

Raloxifene ([6-hydroxy-2-(4-hydroxyphenyl)-1-benzothiophen-3-yl]-[4-(2-piperidin-1-ylethoxy) phenyl]methanone) and analog A (6-methoxy-2-(4-methoxy-phenyl)-3-(3-trifluoromethyl-phenylsulfanyl)-benzo[b]thiophene 1-oxide) were purchased from Sigma (St. Louis, MO, USA). Analog B (1-{4-[2-(1-piperidinyl)ethoxy]phenyl}ethanone oxalate) and analog C ({4-[2-(4-methyl-1-piperidinyl)ethoxy]phenyl}(phenyl)methanone oxalate) were purchased from Chembridge (San Diego, CA, USA). Analog D (2-[4-(4-Chlorobenzoyl)phenoxy]-1-(1-piperidinyl)ethanone) was from Enamine (Monmouth Junction, NJ, USA). Analog E ([6-hydroxy-2-(4-hydroxyphenyl)-1-benzothiophen-3-yl]-[4-(4-propan-2-ylpiperazin-1-yl)phenyl]methanone), also referred to as Y134, was purchased from Tocris (Minneapolis, MN, USA). Analog F ([6-methoxy-2-(4-methoxyphenyl)-1-benzothiophen-3-yl]-[4-(2-piperidin-1-ylethoxy)phenyl]methanone) was purchased from Toronto Research Chemicals (Toronto, ON, Canada). Analog G (3-(4-Fluorophenoxy)-6-methoxy-2-(4-methoxyphenyl)-1-benzothiophene 1-oxide) was purchased from Ryan Scientific (Mount Pleasant, SC, USA).

### 2.4. Luciferase Reporter Assay

AhR reporter gene assays were performed as described previously [17,18,19] with modifications. Briefly, cells transfected with AhR response element (AhRE)-luciferase reporter were seeded in 96-well plates at a density of 10,000 cells per well and treated for 15 h with the indicated compounds or 0.1% DMSO as a vehicle control. To investigate possible antagonism by analogs, cells were pre-treated with the indicated analog for 1 h followed by further incubation for 12 h in the presence of either TCDD, 3-MC, or raloxifene. Cells were lysed in 1× cell culture lysis reagent (Promega, Madison, WI, USA) and luciferase activity was measured using the Luciferase assay system (Promega) according to the manufacturer’s protocol. The luminescence signal was collected using a Tropix TR717 microplate luminometer (Berthold, Bad Wildbad, Germany) and WinGlow software. The reporter gene activity was normalized to vehicle control. 

### 2.5. Cell Viability Assay

Cells were seeded in 96-well plates at specified densities of 2000 to 5000 cells per well, grown overnight, and treated with the indicated compounds or 0.1% DMSO as a vehicle control for 48 h or 72 h. Cell viability was measured using CellTiter GLO assay kit (Promega) according to the manufacturer’s protocol. Luminescence data was collected as described above. 

### 2.6. Real-Time Quantitative PCR

Total RNA was prepared using a total RNA kit (Omega Bio-tek, Norcross, GA, USA). First strand cDNA was synthesized using a Transcriptor kit (Roche, Indianapolis, IN, USA). Real-time qPCR was performed using FastStart Universal SYBR Green master mix (Roche, Indianapolis, IN, USA) and a 7500 Fast PCR system (Applied Biosystems, Foster City, CA, USA) according to the manufacturer’s protocol. The human primer sequences used in this study were as follows: GAPDH forward, 5′-ACCTTTGACGCTGGGGCTGG-3′; GAPDH reverse, 5′-CTCTCTTCCTCTTGTGCTCTTGCTGG-3′; CYP1A1 forward, 5′-CTTCACCCTCATCAGTAATGGTC-3′; CYP1A1 reverse, 5′-AGG CTGGGTCAGAGGCAAT-3′. Relative levels of mRNA expression were determined using a standard comparative Ct method. Data were analyzed as described above.

### 2.7. Western Blotting

Analysis of protein abundance was performed by Western blot, according to standard techniques. Cell lysates were collected using RIPA buffer with protease inhibitors, and were quantified using BCA assay. Some cell lysates were collected using 2× Laemmli buffer directly. Samples were boiled for 5 min, separated by 10% SDS PAGE, and transferred to PVDF membranes by semi-dry transfer. Blots were probed using AhR (BML-SA-210, Enzo Life Sciences, Farmingdale, NY, USA) and GAPDH primary antibodies (sc-365062, Santa Cruz Biotechnology, Dallas, TX, USA). Chemiluminescence signal was developed using horse radish peroxidase conjugated secondary antibodies (SouthernBiotech, Birmingham, AL, USA) and SuperSignal West Pico reagent (Thermo Fisher Scientific, Waltham, MA, USA). Images were captured using a G:BOX imaging system and GeneSys software version 1.5.9 (Syngene, Cambridge, UK).

### 2.8. Zebrafish Embryo Toxicity Assay 

Zebrafish are a vertebrate model system useful for testing both the biological activity and toxicity of small molecules, including new drug leads. Zebrafish larvae have functional organs and express orthologs of the majority of human proteins, and numerous chemicals have been shown to have similar effects between human, rodent, and zebrafish [20]. In addition, zebrafish embryos have been used to assess hazards of numerous chemicals [21,22]. For these reasons, zebrafish are widely employed in the early stages of drug discovery and in selection of pre-clinical compounds with desirable end points [20].

The Institutional Animal Care and Use Committee of Oregon State University approved this protocol (ACUP Number: 4748; August 2016). Tropical 5D strain (T5D) zebrafish were housed at the Oregon State University Sinnhuber Aquatic Research Laboratory. Adults were housed in density of 1000 fish per 100 gallon in standard laboratory conditions of 28 °C on a 14 h light/10 h dark photoperiod in fish water. Embryos were collected and staged as described by Kimmel et al. [23]. Compounds were tested on 5 h post fertilization (hpf) embryos at 0.5 µM, 1.9 µM, 7.1 µM, 27 µM, and 100 µM in 0.64% dimethyl sulfoxide. At 24 and 120 hpf, the effects of compounds on zebrafish embryos were evaluated as reported previously [22,24].

### 2.9. Flow Cytometry

For assessment of apoptosis, cells were first seeded into 6-well tissue culture plates to give approximately 50% confluence and allowed to attach overnight. The cells were then treated for 24 h with the indicated compound. Apoptosis was evaluated using an Annexin V apoptosis detection kit PerCP-eFluor™ 710 (cat#88-8008, Thermo Fisher Scientific, Waltham, MA, USA). Data was acquired using a CytoFLEX S flow cytometer (Beckman Coulter, Brea, CA, USA) comprising 10,000 events on the PC5.5 channel and analyzed with CytExpert software (Beckman Coulter). Cell cycle analysis was performed by ethanol fixation of cells, followed by incubation with 1 µg/mL Hoechst 33258. 

### 2.10. Statistical Analysis

Data were analyzed by one-way ANOVA followed by Dunnett test using GraphPad Prism version 5 (GraphPad Software, La Jolla, CA, USA). *P* values less than 0.05 were regarded as statistically significant. 

## 3. Results

**Figure 1:**
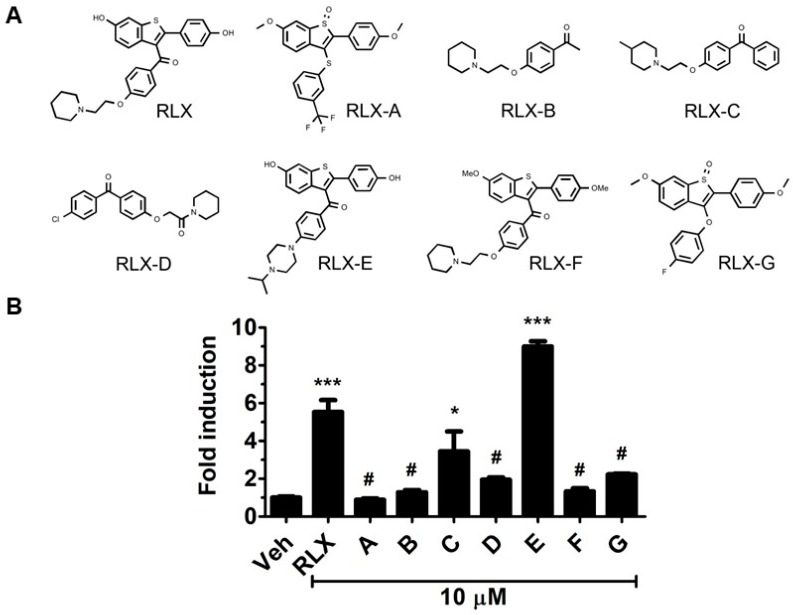
Structural analogs of raloxifene. (**A**) Four analogs share structural similarity to the raloxifene core (A, E, F, and G), and three analogs (B, C, and D) have similarity to the non-core aspects of raloxifene. (**B**) The ability to activate AhR transcriptional activity was measured by AhRE-luciferase reporter activity in Hepa1 cells treated for 15 h with 0.1% DMSO (vehicle) or the indicated compounds. Reporter gene activity is normalized to the vehicle control and is presented as fold-change. Data are presented as the mean ± SEM (*n* = 3–6), and were analyzed by one-way ANOVA followed by Dunnett test. *, *p* < 0.05; ***, *p* < 0.001; #, not significant.

We first investigated the structural aspects of raloxifene needed for AhR activation. Seven analogs of raloxifene were selected and evaluated for their ability to induce AhR transcriptional activity (Figure 1A). Analog B, which is identical to the basic side chain of raloxifene with three possible hydrogen bond acceptors (one nitrogen and two oxygen atoms), failed to produce any significant AhR activation (Figure 1B). On the other hand, analog C activated the AhR by approximately three-fold, which was possibly attributable to the additional phenyl ring and/or the methyl group on the piperidine ring. Analog D did not significantly induce AhR-driven reporter gene expression, which together with analogs B and C suggested that the piperidine containing moiety alone may not be sufficient for activation of the AhR. We next tested four additional raloxifene analogs A, E, F, and G, which share the benzothiophene core. We found that analog E, also referred to as Y134, increased AhR activation (Figure 1B). 

**Figure 2:**
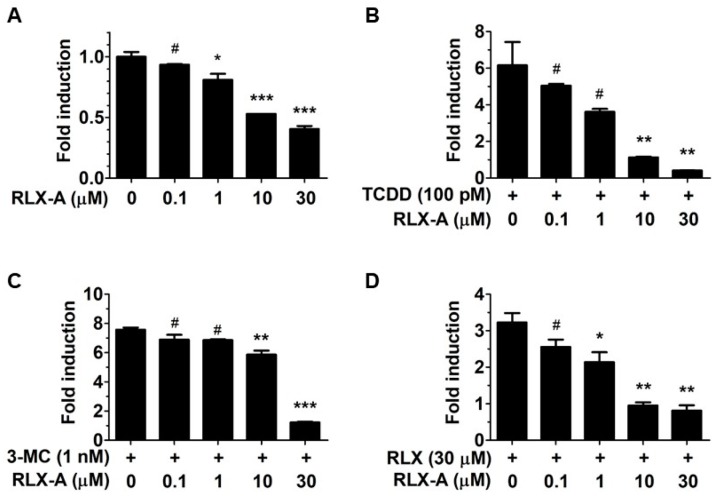
Analog A antagonizes AhR activation. The antagonistic activity of analog A was analyzed by AhRE-luciferase reporter assay. (**A**) Hepa1 cells transfected with AhRE were incubated for 12 h with analog A or vehicle as indicated and the luciferase reporter activity was measured by Luciferase assay system. The data was normalized to cell viability, which was determined with CellTiter GLO assay performed simultaneously. (**B**–**D**) Cells were pre-incubated for 1 h with 0.1% DMSO or analog A at the indicated concentrations (0.1 µM, 1 µM, 10 µM, and 30 µM) followed by treatment for 12 h with 100 pM TCDD (**B**), 1 nM 3-methylcholanthrene (**C**), or 30 µM raloxifene (**D**). The data are presented as fold inductions, and are the mean ± SEM (*n* = 2). Data were analyzed by one-way ANOVA followed by Dunnett test’s post-hoc test. Differences with *p* values less than 0.05 was regarded as statistically significant. *, *p* < 0.05; **, *p* < 0.01; ***, *p* < 0.001; #, not significant.

We next asked if any of the compounds that failed to activate the AhR were capable of acting as AhR antagonists and suppressing agonist-induced AhR activation. We found that analog A suppressed the basal level of AhR activation (Figure 2A). Further, analog A inhibited AhR activation by other AhR agonists 2,3,7,8-tetrachlorodibenzo-p-dioxin (TCDD), 3-methylcholanthrene (3-MC), and raloxifene (Figure 2B–D), whereas analogs B, C and D had little to no effect on AhR activation. The apparent antagonist activity of analog A was not due to cytotoxicity, because analog A did not significantly inhibit cell viability except at the highest concentration of 30 µM. Further, the AhR activation data was normalized to relative cell viability.

**Figure 3:**
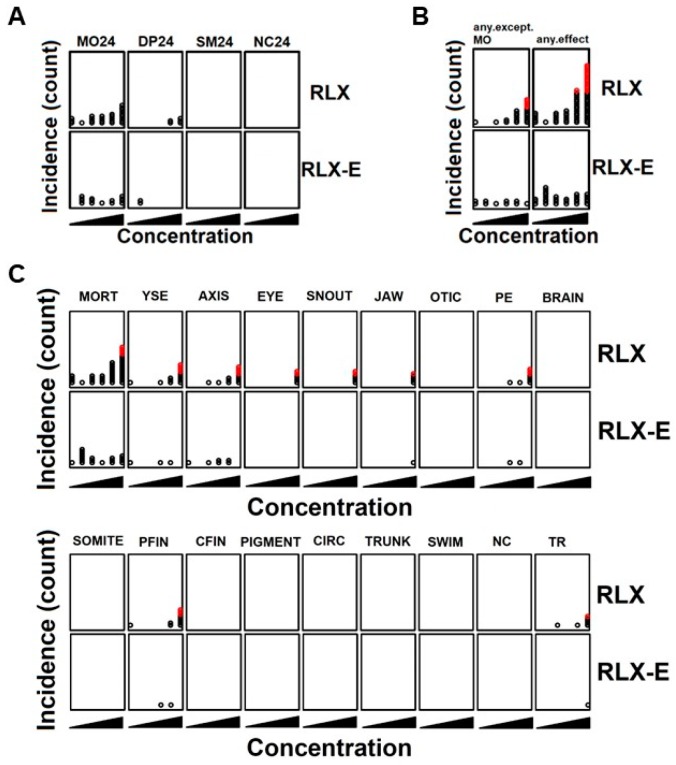
Zebrafish toxicity. (**A**–**C**) To identify potential in vivo toxicity in the developing animal model, zebrafish embryos were treated with raloxifene or analog E/Y134 (vehicle, 0.5 µM, 1.9 µM, 7.1 µM, 27 µM, and 100 µM) at 5 h post fertilization (hpf). Developmental defects and mortality were assessed at 24 hpf (**A**) and 120 hpf (**B**,**C**). Each circle corresponds to the affected embryo, and red circles indicate the incidence of statistical significance suggesting toxicity. MO24, mortality at 24 hpf; DP24, developmental progression at 24 hpf; SM24, spontaneous movement at 24 hpf; NC24, notochord at 24 hpf; any except MO, any effect except mortality at 120 hpf; any effect, anything including mortality at 120 hpf; MORT, mortality at 120 hpf; YSE, yolk sac edema at 120 hpf; AXIS, crooked tails at 120 hpf; EYE, eye swelling at 120 hpf; SNOUT, snout malformation at 120 hpf; JAW, jaw malformation at 120 hpf; OTIC, otic vesicle at 120 hpf; PE, pericardial edema at 120 hpf; BRAIN, brain at 120 hpf; SOMITE, somite at 120 hpf; PFIN, pectoral fin at 120 hpf; CFIN, caudal fin at 120 hpf; PIGMENT, pigmentation at 120 hpf; CIRC, circulation at 120 hpf; TRUNK, trunk at 120 hpf; SWIM, swim bladder at 120 hpf; NC, notochord at 120 hpf; TR, touch response at 120 hpf.

We next evaluated the effect of raloxifene and analog E (Y134) on developing zebrafish embryos to evaluate their relative toxicities in anticipation of future clinical testing as AhR-targeted therapeutics. Compounds were added to zebrafish embryos at 5 h post fertilization (hpf) and their effects on development and survival were assessed at 24 hpf and 120 hpf. The number of affected embryos was used as an indicator of toxicity, and thus any concentration that produced an increased number of affected embryos was regarded as toxic. At 24 hpf, neither raloxifene nor analog E showed significant toxicity (Figure 3A). At 120 hpf, analog E did not exhibit any significant toxicity or mortality while raloxifene was associated with some toxicity at higher concentrations (100 µM), especially pertaining to development (Figure 3B). Specifically, the toxic effects that were associated with 100 µM raloxifene were yolk sac edema (YSE), crooked tails (AXIS), eye swelling (EYE), snout deformity (SNOUT), jaw malformation (JAW), pericardial edema (PE), and pectoral fin malformation (PFIN) (Figure 3C). In addition, raloxifene, but not analog E, inhibited the touch response (TR) partly due to the developmental defects (Figure 3C). Together, these results showed that analog E has a significantly better safety profile compared to equivalent concentrations of raloxifene.

**Figure 4:**
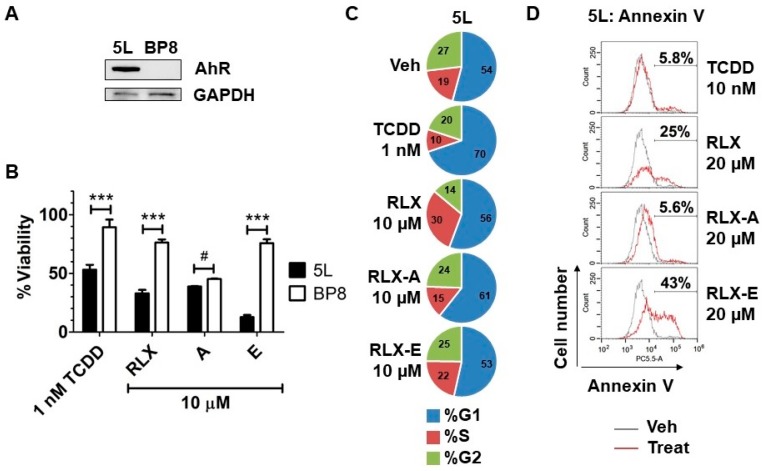
Inhibition of hepatoma cell viability by raloxifene analogs. (**A**) Western blot showing the relative AhR expression in 5L and BP8 cells. (**B**) 5L and BP8 cells were treated with the compounds as indicated, or with 0.1% DMSO for 72 h. Cell viability of the vehicle control was set to 100% and the relative viability is shown as the mean ± SEM (*n* = 3). Two-way ANOVA and Bonferroni post-test were performed to analyze the data for statistical significance ***, *p* < 0.001; #, not significant (**C**) Cell cycle analysis of 5L cells treated with vehicle, 1 nM TCDD, 10 µM raloxifene, 10 µM analog A, or 10 µM analog E for 48 h. (**D**) Apoptosis analysis by flow cytometry. Annexin V staining of 5L cells after 24 h treatment with vehicle, 10 nM TCDD, 20 µM raloxifene, 20 µM analog A, 20 µM analog E. The Annexin V positive population is presented as a percent ratio minus the vehicle control, which was 5.1%.

We previously showed that raloxifene decreases cancer cell viability by inducing apoptosis in an AhR-dependent manner. Given that analog E activates the AhR to a similar degree as raloxifene but with much less toxicity, we next asked if analog E could also induce AhR-dependent inhibition of cell growth. We evaluated the anti-proliferative effects of raloxifene and analog E on hepatoma cells. 5L cells expressing the AhR and the derivative AhR deficient BP8 cells (Figure 4A) were treated with raloxifene or analogs A and E for 72 h, after which the number of viable cells was determined. Analog E decreased the viability of 5L cells but not that of BP8 cells, similar to TCDD and raloxifene (Figure 4B). Analog A reduced viability of both 5L and BP8 cells equally, indicating AhR independent effects. Together, these data suggested that analog E, which is well tolerated in zebrafish embryos, also has AhR-dependent anti-cancer effects. To determine if the reduction of viability was due to cell cycle arrest or induction of apoptosis, we next performed cell cycle analysis and Annexin V staining in 5L cells, respectively. As expected, cell cycle analysis showed that TCDD induced G1 arrest in 5L cells [10,25]. However, raloxifene increased the percentage of cells in the S phase, while analog E did not alter the cell cycle profile compared to vehicle treatment (Figure 4C). According to Annexin V analysis, analog E was a more potent inducer of apoptosis compared to raloxifene in 5L hepatoma cells (Figure 4D). Specifically, after treatment of cells for 24 h with raloxifene or analog E, we found that 25% and 43% of cells were positive for Annexin V staining, respectively, suggesting that analog E induced apoptosis to a more significant degree than the parent molecule raloxifene (Figure 4D). Interestingly, neither analog A nor TCDD induced significant apoptosis compared to vehicle. Taken together, these data suggest that the reduced cell viability seen in AhR expressing 5L hepatoma cells treated with analog E was most likely due to induction of apoptosis.

**Figure 5:**
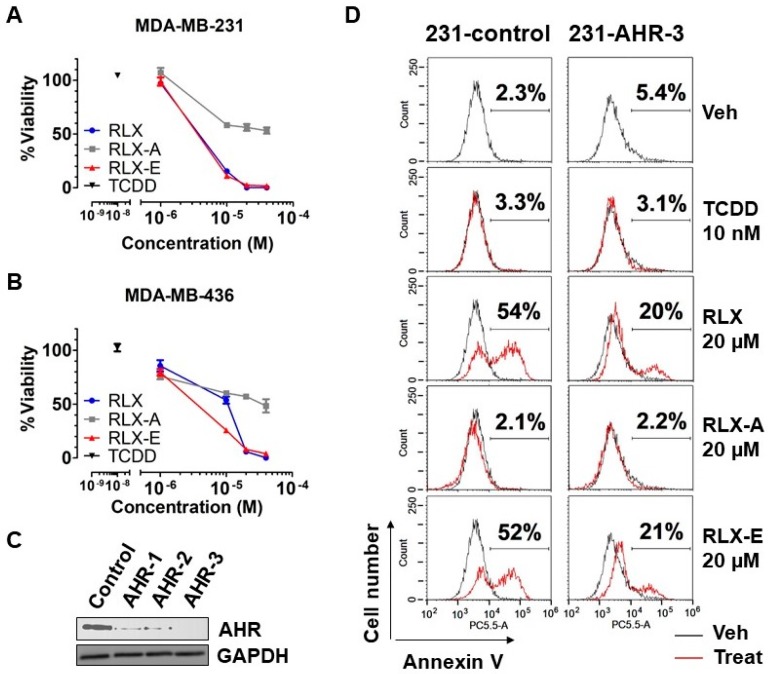
AhR-dependent induction of apoptosis in triple negative breast cancer (TNBC) cells. (**A**,**B**) Human TNBC cell lines MDA-MB-231 and MDA-MB-436 were treated as indicated for 48 h in 1% serum media, followed by analysis for cell viability. The vehicle control was set to 100% and relative viability is shown as the mean ± SEM (*n* = 3). Data were analyzed by two-way ANOVA and Bonferroni post-test. (**C**) Western blot analysis of AhR expression in MDA-MB-231 cells stably transfected with either control vector (control) or AhR guide RNA-expressing pLentiCRISPR vectors (AHR-1, AHR-2, and AHR-3) to confirm suppression of the AhR expression. (**D**) Anexin V staining of MDA-MB-231 cells with (231-control) or without (231-AHR-3) AhR expression. Cells were treated for 24 h with vehicle, 10 nM TCDD, 20 µM raloxifene, 20 µM analog A, or 20 µM analog E in 1% serum media and stained using Annexin V as a marker of apoptosis. The Annexin V positive population was interpreted as apoptotic cells, and the percentage ratio of Annexin V positive cells to the total cell population is shown.

Having shown that treatment of hepatoma cells with raloxifene and analog E led to reduced cell viability and induction of apoptosis, we next investigated the effects of analog E on human ER-negative/AhR-positive breast cancer cells, which we have previously shown to respond to raloxifene in an AhR-dependent manner. We further focused on triple-negative breast cancer (TNBC) based on both the present lack of effective treatment options and poor prognosis of patients [26]. To this end, we selected the TNBC cell lines MDA-MB-231 and MDA-MB-436 based on their AhR expression profile. Both MDA-MB-231 and MDA-MB-436 cells were treated with TCDD, raloxifene, or analogs A and E for 48 h, after which the number of viable cells was determined. Raloxifene and analog E both reduced the viability of MDA-MB-231 cells at a concentration of 10 µM (Figure 5A). Similarily, raloxifene and analog E both reduced the viability of MDA-MB-436 cells. However, treatment of MDA-MB-436 cells with analog E at 10 µM resulted in a 75% reduction in viable cells compared with a 47% reduction with raloxifene treatment (Figure 5B). In addition, analog A reduced the viability of both MDA-MB-436 and MDA-MB-231 cells by 50% at 40 µM, the highest concentration tested (Figure 5A,B). Data presented in Figure 4B suggested that the effects of analog A are independent of AhR expression. On the other hand, treatment of the TNBC cell lines with 10 nM TCDD, a concentration known to maximally activate AhR transcription and Cyp1a1, one of the most well-known and studied target genes of the AhR [27,28], had no significant effect on cell viability compared to vehicle (Figure 5A,B). 

In order to determine if the reduced viability of human TNBC cells treated with analog E was mediated through the AhR, we next generated MDA-MB-231 AhR knockout lines using CRISPR. Western blot analysis of three derived lines indicated that the MDA-MB-231 AHR-3 line did not express AhR protein (Figure 5C). Therefore, MDA-MB-231 AHR-3 (231-AHR-3) cells were selected to evaluate the AhR dependence of analog E with respect to induction of apoptosis. Control and 231-AHR-3 lines were treated with raloxifene and analogs A and E for 24 h followed by Annexin V analysis using flow cytometry. We found that treatment of the AhR expressing control line (231-control) with raloxifene or analog E led to 54% and 52% of cells staining positively for the apoptosis marker Annexin V, respectivly. Conversely, the degree of annexin V staining induced by the raloxifene and analog E was significantly diminished to approximatly 20% and 21% in the 231-AHR-3 AhR knockout cell lines, respectivly (Figure 5D). Consistent with our data thus far, neither TCCD nor the transcriptionally inactive analog A increased Annexin V staining in cells with or without AhR expression (Figure 5D). Taken together, these data demonstrated that raloxifene and analog E/Y134 induce apoptosis in human TNBC cells in an AhR-dependent manner. 

## 4. Discussion

We recently showed that the selective estrogen receptor modulator raloxifene is a new AhR ligand that is effective in inducing AhR-mediated transcriptional activity and inhibiting tumor cell growth in an AhR dependent manner [14]. In this study, we characterized seven structural analogs of raloxifene for their ability to activate the AhR. Specifically, we evaluated these compounds for their ability to induce AhR-mediated gene transcription and inhibit cancer cell growth through a mechanism requiring AhR expression. Both raloxifene and analog E inhibited the growth of TNBC cells and induced apoptosis in an AhR-dependent manner. Together, these data suggest that the anti-cancer effects of raloxifene analog E are similar to those of the parent raloxifene molecule. 

Raloxifene is well tolerated in patients, where it is used for prevention of estrogen receptor positive breast cancer and for the treatment of osteoporosis. However, in the context of using raloxifene or analog E as AhR-targeted therapeutic, we also investigated the toxicity profiles of these moleules using the well-established preclinical zebrafish platform [20,23]. While we found that raloxifene was toxic to zebrafish embryos at high concentrations (100 µM; Figure 3), analog E had no toxicity at the same concentration. In addition, analog E had better AhR-dependent effects in hepatoma cells compared to raloxifene (Figure 4). 

Similar to raloxifene, analog E/Y134, was initially characterized as a selective estrogen receptor modulator (SERM) [29]. Interestingly, analog E was shown to inhibit the growth of not only ER-positive breast cancer cells, but also ER-negative MDA-MB-231 cells at concentrations of 10 µM or higher [30]. In the present study, we found that analog E inhibited hepatoma cell growth in an AhR dependent manner at a concentration of 10 µM (Figure 4B). Furthermore, we demonstrated that analog E induces apoptosis in an AhR dependent manner in MDA-MB-231 cells (Figure 5D). Thus, our findings suggest that analog E inhibits growth of ER-negative breast cancer cells via a previously unappreciated activation of the AhR.

Analog E differes structurally from raloxifene with respect to the location of a 1-isopropylpiperazine side chain in place of a piperidine moiety, suggesting that this basic side chain may be a useful target for optimization to identify novel AhR ligands with improved anti-cancer efficacy. On the other hand, the piperidine-containing non-benzothiophene core was inactive with respect to AhR transcription (Figure 1B), suggesting that the benzothiophene core (2-(4-hydroxyphenyl)benzo[b]thiophene-6-ol) may be necessary but not sufficient for AhR activation. Interestingly, raloxifene analog F, a raloxifene bismethyl ether, also failed to induce AhR activation (Figure 1B), suggesting that the presence of one or both methyl groups on the benzothiophene core may weaken AhR activation. 

Previous computational modeling work has shown the that 6-hydroxyl group on the benzothiophene ring of raloxifene forms hydrogen bond with the backbone carbonyl groups of the AhR residues I349 and V363 [14]. Thus, the methyl ether on the benzothiophene ring of analog F may break the hydrogen bond, subsequently decreasing AhR activation. In future studies, it will be interesting to compare the activity of analog E with that of Arzoxifene (LY 353381), which has been shown previously to prevent breast cancer in rat models [31], and is predicted to have similar AhR activation as raloxifene owing to the presence of the 6-hydroxyl group on the benzothiophene ring. Additional small molecules that will be of interest to explore in the context of AhR-activation include LY 117018, which has five-membered heterocyclic pyrrolidine ring in place of the six-membered piperidine ring of raloxifene [15]. Likewise, studies on TZE-5323, in which the 4-hydroxyphenyl group on the benzothiophene of raloxifene is replaced with a cyclohexyl group [32], will help to determine the contribution of the 4-hydroxyphenyl group on AhR activation.

A final aspect of this study was the identification of raloxifene analog A as a partial antagonist of the AhR capable of inhibiting AhR driven reporter expression by TCDD or 3-MC. Thus, analog A may be useful for designing AhR antagonists that better modulate AhR transcriptional activity in order to obtain desirable biological outcomes such as increasing hematopoietic stem cell populations [33].

## 5. Conclusions

In conclusion, we showed that raloxifene analogE/Y134 is an AhR agonist with AhR-dependent anti-cancer effects. Y134 induces AhR-dependent cell death in TNBC cells. We also identified raloxifene analog A as a partial antagonist of the AhR. Consistent with our previous report [12], our finding suggests that raloxifene-based compounds have the potential to be developed as AhR selective anti-cancer agents.
